# Maternal gastrointestinal nematode infection enhances spatial memory of uninfected juvenile mouse pups

**DOI:** 10.1038/s41598-022-13971-y

**Published:** 2022-06-13

**Authors:** Sophia C. Noel, Liana Fortin-Hamel, Manjurul Haque, Marilyn E. Scott

**Affiliations:** Institute of Parasitology, McGill University (Macdonald Campus), 21,111 Lakeshore Road, Ste-Anne de Bellevue, Quebec, H9X 3V9 Canada

**Keywords:** Spatial memory, Behavioural ecology, Parasitic infection, Disease model

## Abstract

The developing brain is particularly vulnerable to factors including maternal infection during pregnancy. Establishment of neural networks critical for memory and cognition begins during the perinatal period, when *Heligmosomoides bakeri*, a gastrointestinal (GI) nematode restricted to the maternal mouse intestine, has been shown to upregulate expression of long-term potentiation genes in the young rodent pup brain. We explored the impact of maternal infection during pregnancy and early lactation on the spatial behavior of uninfected male and female juvenile mice. Pre-weaned pups of *H. bakeri* infected dams exhibited less exploratory behaviour compared to pups of uninfected dams on postnatal day (PD) 16 but not PD 17, possibly reflecting a transient fear of an unfamiliar environment and/or a brief neurodevelopmental delay. Our two spatial memory tests show for the first time an enhancement of spatial memory in response to maternal nematode infection regardless of pup sex. At PD 17, pups of infected dams expressed object location memories after 3 h in the Object Location Test whereas offspring of uninfected mothers did not. In addition, at PD 34, juveniles of infected mothers retained their ability to find the escape hole in the Barnes Maze Test for one week whereas offspring from uninfected mothers did not. This finding is even more striking given that spatial memory was positively associated with pup length, yet this maternal infection impaired linear growth of pups. Thus, the positive impact of maternal infection on spatial memory countered any impairment associated with the shorter length of the pups. Overall, these novel findings indicate that a maternal GI nematode infection during pregnancy and lactation positively influences the spatial memory of uninfected juvenile offspring with potential fitness implications for the next generation.

## Introduction

In most natural environments, terrestrial mammal populations harbour gastrointestinal (GI) helminths that often live as adults for prolonged periods^1^. Parasites have been found to have profound effects on host behaviour and cognition^2,3^, and the impact of GI helminths on cognitive function has been debated for many years^4^. Rodent studies in controlled laboratory settings showed that the GI helminths *Nippostrongylus brasiliensis*^4^*, Heligmosomoides polygyrus*^5^ and *Ancylostoma ceylanicum*^6^ impaired spatial learning and memory of the infected host, that *Hymenolepsis diminuta* improved spatial memory of the infected host^7^, and that *Strongyloides ratti* did not influence spatial learning or memory of the infected host^8^. Spatial memory is an important aspect of cognitive function that is needed both to plan a route to a desired location and to remember where an object is located or where an event occurred^9^. For mammals, mate location, foraging, predator avoidance and territorial defence are all dependent on spatial memory, and it is therefore an essential aspect of survival^10^. Impairment of spatial memory may thus reduce fitness of the infected host, while an enhancement would assumingly be beneficial. Previous studies have focused on the infected host, but it is unknown whether maternal GI helminth infection influences the spatial behaviour of the next generation. This is surprising as pregnancy increases the risk of helminth infection^11,12^, and brain development has been shown to be particularly vulnerable to factors such as maternal stress, malnutrition, and infection during pregnancy^13–15^.

Brain development is an extremely complex and sensitive process which begins during the intrauterine period and continues postnatally in rodents until three-months of age when brain maturation is completed^16^. Active exploration of space begins in the second week of life^17^, but the refinement and maturation of neural circuitries necessary for efficient processing of spatial cognition only occurs at three to four weeks of age^17^ when rodents can form and retain spatial memories for the location of objects and the route to a specific location^18,19^. During this developmental period, the persistent strengthening of synapses that produces a long-lasting increase in signal transmission between two neurons is an important and necessary process for spatial memory formation^20^. This process is called long-term potentiation (LTP), and occurs in all excitatory pathways in the hippocampus^20^, the part of the mammalian forebrain network that is necessary for spatial cognition^21^. As functional hippocampal memory is sensitive to perturbation^22^, any stressors that occur during the neurodevelopmental period, including maternal infection, can have long-lasting consequences on brain function and behaviour. For example, exposure of pregnant rodents to *Escherichia coli*^23,24^ or influenza virus^25^ reduced the induction of hippocampal LTP in offspring, and impaired their spatial exploration, learning and memory.

Among the behavioral tasks designed to assess spatial behaviours in rodents^9^, the Open Field Test (OFT) is commonly used to assess exploratory behaviour. Spontaneous exploration is first detectable between post-natal day (PD) 16–19 and a lack of exploration may indicate anxiety^17,26^. For spatial memory assessment in young mice, the Object Location Test (OLT) is minimally stressful and relies on an animal's intrinsic preference for novelty^27^. This test is hippocampus-dependent and assesses the ability of rodents to recognize that the location of an object has changed between a training and test trial, evidenced by an increase in investigation of the object after it has been displaced^27,28^. Young mice (PD 16–17) can retain object location memories for 1–10 min^18,29^ but longer-term retention does not occur until PD 21–24^18,30^. The Barnes Maze Test (BMT) is another spatial test that avoids the use of strong aversive stimuli, and assesses hippocampus-dependent spatial reference memories formed over repeated trials in an unchanging environment^9^. This test assesses the ability of rodents to learn and recall the location of an escape box which is located under one of 20 holes around the perimeter of a platform^9^. The BMT can assess both short-term spatial reference memory one day after the training phase and long-term spatial reference memory one week later^31^. Rodents are capable of learning the route to an escape location at PD 21–23^17,19,32,33^, but the ability to retain long-term memories for this location does not normally occur until adulthood^32,33^.

In examining the impact of maternal infection on offspring cognition, it is important to recognize that both offspring sex^34–37^ and body length^38–41^ may influence the results. The ability of male rodents to outperform females in spatial tasks may be linked to hormonal influences^37,42^, or the size of the hippocampus^43^. Height is a strong indicator of brain size and cognitive function, including memory, in humans^38–41^. In rodents, length has shown to be associated with brain size^44^, which is associated with cognition^45^. Although a relationship between length and cognitive performance has not been documented in rodents, it may be important to control for length when measures of brain size are not available especially as maternal infections can impair offspring linear growth^46,47^.

Due to the gap in research surrounding the influence of a maternal GI helminth infection on the neurodevelopment of offspring, a recent study has explored the consequences of maternal infection with the GI nematode, *Heligomosomoides bakeri* (also referred to as *Heligomosomoides polygyrus* and previously known as *Nematospiroides dubius*), on neonatal brain development^48^. *H. bakeri* is common in wild mouse populations with a prevalence as high as 86%^49^, and is a commonly used laboratory model^50^. This strictly intestinal parasite has a direct lifecycle whereby eggs shed in the feces of the infected mouse hatch in the external environment and undergo two molts to become infective third stage larvae (L_3_) within 7 days. Infective L_3_ are then ingested and penetrate the submucosa of the small intestine before returning to the intestinal lumen as adult worms where they mate, and female worms release eggs^50^. Infected mice mount a strong type 2 (Th2) immune response against the parasite, however, adult worms are capable of stimulating an immunoregulatory network (Treg) which facilitates parasite survival^50,51^. *H. bakeri* infection of pregnant and lactating mice has been shown to alter gene expression in fetal^52^ and neonatal brains^48^, and to up-regulate five key interacting pathways associated with LTP in uninfected one-week old male pups^48^. These findings raise the intriguing hypothesis that a maternal *H. bakeri* infection may improve synaptic plasticity, cognition and memory of the next generation. The goal of this study was to explore the influence of maternal *H. bakeri* infection on the spatial behaviour of uninfected pre-weaned and juvenile male and female offspring.

## Results

This study assessed the influence of maternal *H. bakeri* infection on the spatial behaviour of uninfected male and female juvenile offspring. Outbred CD-1 mice were infected repeatedly or sham infected during pregnancy and lactation and litters from 8 uninfected and 8 *H. bakeri* infected dams were used to explore spatial exploration, learning and memory as well as litter size, crown-rump length, and body mass.

### Impact of maternal infection on litter size

There was no significant effect of maternal infection on litter size (uninfected: 12.4 ± 1.12; infected: 12.9 ± 0.61; P = 0.7).

### Impact of maternal infection and offspring sex on pup crown-rump length and body mass

Pups born to infected dams had shorter length and lower mass than pups of uninfected dams, at both PD 15 and 21 (all P values < 0.0001, Supplementary Fig. S1). In addition, male pup length and mass were larger than female pups at both PD 15 and 21 (all P values < 0.005, Supplementary Fig. S1).

### Impact of maternal infection on offspring spatial behavior

#### Early exploratory behavior

In the OFT, pups of infected dams exhibited less spatial exploration at PD 16 than pups of uninfected dams as evidenced by lower *total path traveled* (P = 0.01)*, mean velocity* (P = 0.01), and *time in center zone* (P = 0.015) and a greater *time without movement* (P = 0.006) (Table 1). There were no sex effects (all P values > 0.15, data not shown).

**Table 1:** Effect of maternal *H. bakeri* infection on displacement variables measured during the 10 min Open Field Test.

Variable	Pups of uninfected dam	Pups of *H. bakeri* dam	Test statistic and P value
Total path traveled (cm)	2586 ± 354	1191 ± 353	*x*^2^_1_ = 6.70; P = 0.01
Mean velocity (cm/s)	4.3 ± 0.6	2.0 ± 0.6	*x*^2^_1_ = 6.69; P = 0.01
Time without movement (%)	51.1 ± 5.4	74.3 ± 5.4	*x*^2^_1_ = 7.70; P = 0.006
Time in center zone (%)	11.4 ± 2.1	4.3 ± 1.6	*x*^2^_1_ = 5.96; P = 0.015

Pups were nested within dam and pup crown-rump length was included as a covariate. Since no significant sex differences were evident, pooled female and male data are shown. Values are LSmeans ± SEM of each outcome variable, n = 32 pups from 8 uninfected dams and n = 32 pups from 8 infected dams.

#### Object location memory

**Figure 1:**
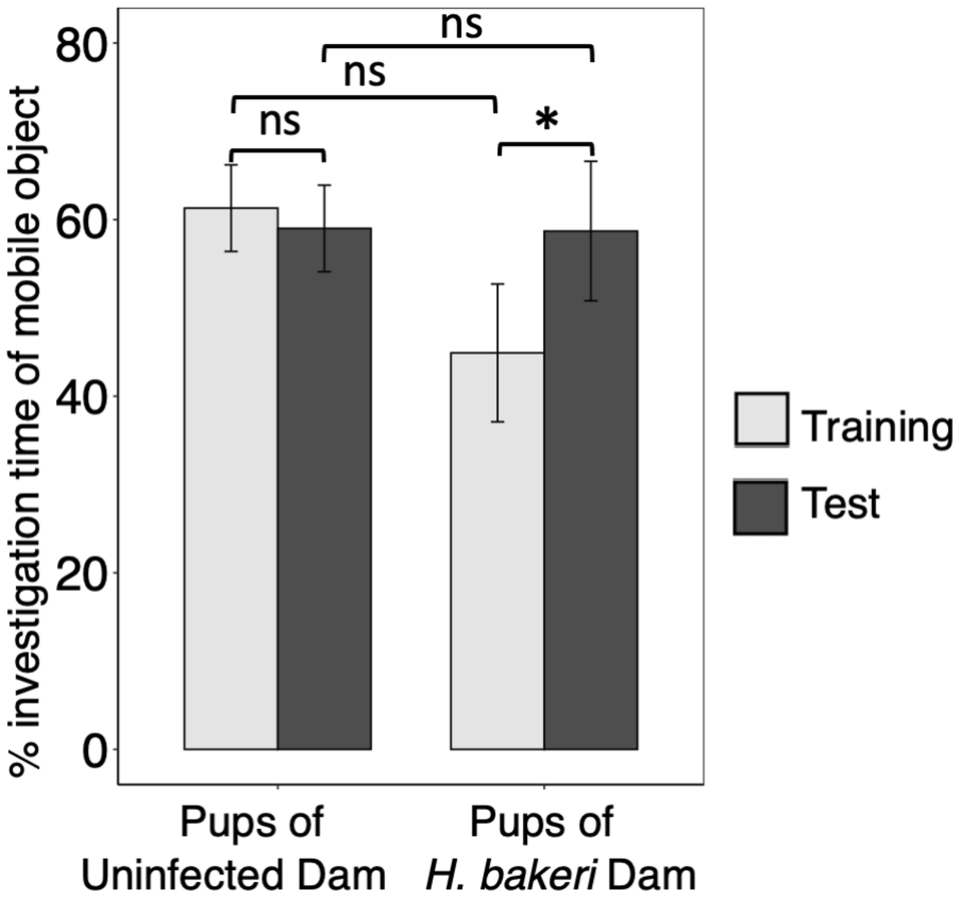
Effect of maternal *H. bakeri* infection on % investigation of mobile object in the training trial, in the test trial, and between the training and test trials in the Object Location Test. In all models, pups were nested within dam, and pup crown-rump length was included as a covariate. Since no significant sex differences were evident, pooled female and male data are shown. Values are means ± SEM, n = 24 pups from 8 uninfected dams and n = 18 pups from 7 infected dams (*ns* not significant, *P < 0.05).

There was no bias in exploration of Objects 1 and 2 during the training phase (P = 0.40). Furthermore, maternal infection did not affect the *total path traveled, mean velocity, time without movement*, *object investigation time* (Table 2) or *% investigation time of mobile object* (Fig. 1) during the training trial or during the test trial. Additionally, there were no sex effects (all P values > 0.27, data not shown).

**Table 2:** Effect of maternal *H. bakeri* infection on displacement and exploration variables measured during the 5 min Training and Test trials of the Object Location Test.

Variable	Pups of uninfected dam	Pups of *H. bakeri* Dam	Test statistic and P value
**Training trial**			
Total path traveled (cm)	1378 ± 192	1766 ± 230	*x*^2^_1_ = 1.37; P = 0.24
Mean velocity (cm/s)	4.6 ± 0.6	5.9 ± 0.8	*x*^2^_1_ = 1.36; P = 0.24
Time without movement (%)	52.0 ± 5.6	44.5 ± 6.6	*x*^2^_1_ = 0.61; P = 0.44
Object 1 (stationary) investigation (s)	4.4 ± 1.1	5.8 ± 1.3	*x*^2^_1_ = 58; P = 0.44
Object 2 (mobile) investigation (s)	6.1 ± 1.4	4.4 ± 1.6	*x*^2^_1_ = 0.52; P = 0.47
Total investigation of both objects (s)	10.6 ± 1.8	10.6 ± 2.2	*x*^2^_1_ = 0.0001; P = 0.997
**Test trial**			
Total path traveled (cm)	1391 ± 254	1564 ± 297	*x*^2^_1_ = 0.16; P = 0.69
Mean velocity (cm/s)	4.7 ± 0.9	5.2 ± 1.0	*x*^2^_1_ = 0.15; P = 0.70
Time without movement (%)	53.4 ± 5.5	49.5 ± 6.6	*x*^2^_1_ = 0.17; P = 0.68
Object 1 (stationary) investigation (s)	4.9 ± 1.0	3.5 ± 1.1	*x*^2^_1_ = 0.70; P = 0.40
Object 2 (mobile) investigation (s)	6.5 ± 2.2	6.8 ± 2.7	*x*^2^_1_ = 0.01; P = 0.94
Total investigation of both objects (s)	11.4 ± 2.5	10.4 ± 3.0	*x*^2^_1_ = 0.05; P = 0.81

Pups were nested within dam and pup crown-rump length was included as a covariate. Since no significant sex differences were evident, pooled female and male data are shown. Values are LSmeans ± SEM of each outcome variable, n = 24 pups from 8 uninfected dams and n = 18 pups from 7 infected dams.

**Figure 2:**
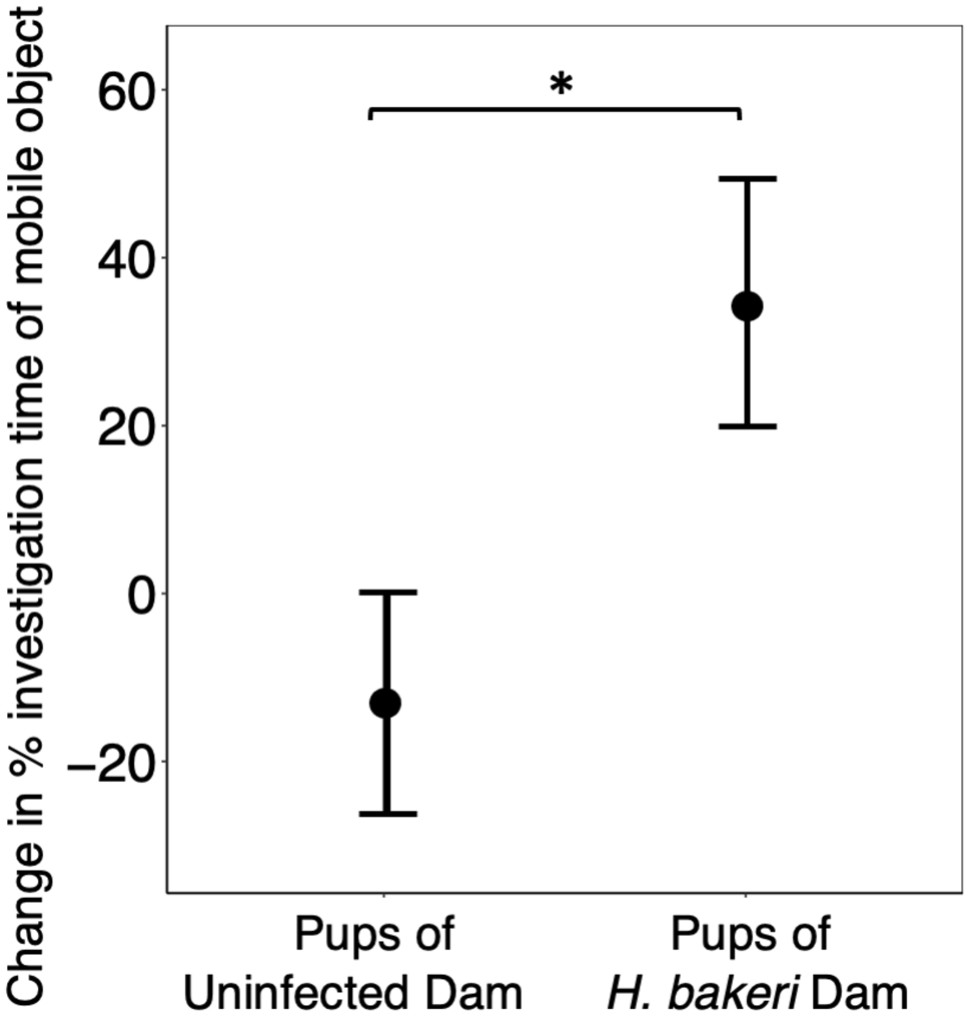
Effect of maternal *H. bakeri* infection on offspring change in % investigation time of mobile object between test and training trial of the Object Location Test. A positive value indicates increased investigation of the object that had been moved between the training and test trial, representing expression of object location memory. Pups were nested within dam, and pup crown-rump length was included as a covariate. Since no significant sex differences were evident, pooled female and male data are shown. Values are LSmeans ± SEM, n = 24 pups from 8 uninfected dams and n = 18 pups from 7 infected dams (*P < 0.05).

However, when using our derivative variables to compare object location memory between training and test trials, we found that maternal infection improved pup memory. Comparing the *% investigation time of mobile object* between training and test trials, pups born to infected dams remembered object locations and explored the moved object significantly more during the test trial compared to the training trial (P = 0.019) (Fig. 1) whereas pups of uninfected dams spent a similar % *investigation time of mobile object* in both the training and test trial (P = 0.74) indicating that they had not retained object location memory after a 3 h period (Fig. 1). This was also reflected in the *change in % investigation time of mobile object* (Fig. 2). Pups of infected dams increased their investigation of the mobile object during the test trial whereas pups of uninfected dams did not, and the difference between these two groups was significant (P = 0.027), providing further evidence that pups of infected dams were able to recall object location memory for 3 h. These findings were not affected by offspring sex (all P values > 0.28, data not shown), but *change in % investigation time of mobile object* was positively associated with pup length (P = 0.041, data not shown), independent of maternal infection.

#### Spatial learning

**Figure 3:**
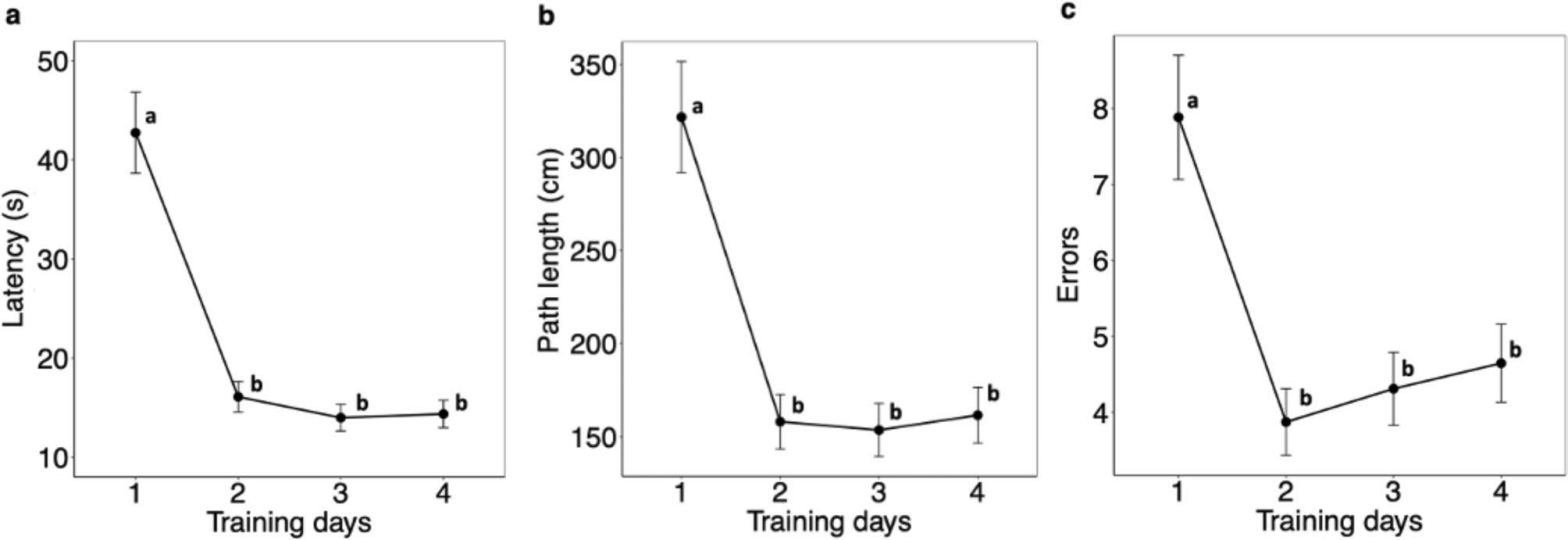
Spatial learning in the Barnes Maze Test over four training days. The identity of the pups was nested within dam for statistical analysis, and pup crown-rump length was included as a covariate. Since no significant differences were evident as a result of maternal treatment condition or offspring sex, pooled data is shown. Values are LSmeans ± SEM, n = 64 pups from 16 dams. (**a**) latency, (**b**) path length and (**c**) errors to reach the escape hole. Means without a common letter differ significantly, P < 0.05.

In the BMT, regardless of maternal infection or offspring sex, pups learned the location of the escape hole on the first training day as indicated by a decrease in the average *latency* (P < 0.0001; Fig. 3a), *path length* (P < 0.0001; Fig. 3b), and *errors* (P < 0.0001; Fig. 3c) between training days 1 and 2. Thereafter, values remained low. Neither maternal infection nor offspring sex influenced *mean velocity* (all P values > 0.27, data not shown).

#### Short and long-term spatial reference memory

**Figure 4:**
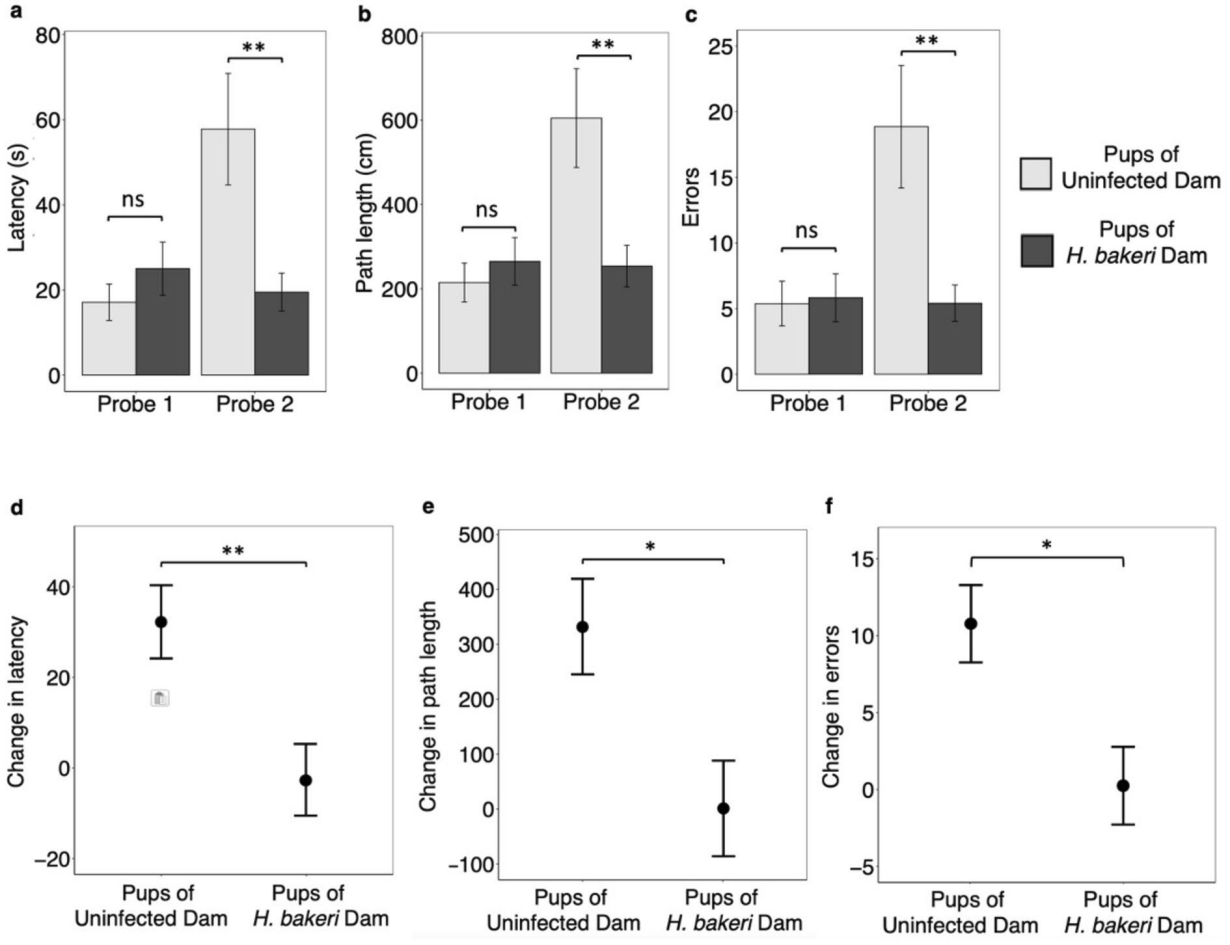
Effect of maternal *H. bakeri* infection on offspring short-term (probe trial 1) and long-term (probe trial 2) spatial reference memory and change in reference memory between probe trials 1 and 2 in the Barnes Maze Test. Probe trial 1 was conducted 24 h after the last training day and probe trial 2 was conducted one-week later. Graphs A, B and C compare juveniles of uninfected vs *H. bakeri* infected dams within probe trial 1 and 2. Graphs D, E and F show the change in performance between probe trials 1 and 2 calculated by subtracting the value in probe trial 1 from the value in probe trial 2 for each mouse. A value of zero or a negative value would indicate that the subject performed as well or better during probe trial 2 compared to probe trial 1, suggesting strong memory retention. (**a**) latency, (**b**) path length, (**c**) errors to reach the escape hole, (**d**) change in latency, (**e**) change in path length and (**f**) change in errors*.* Pups were nested within dam, and pup crown-rump length was included as a covariate. Since no significant sex differences were evident, pooled female and male data are shown. Values are LSmeans ± SEM, n = 32 pups from 8 uninfected dams and n = 32 pups from 8 infected dams (*ns* not significant, *P < 0.05, **P < 0.01).

Neither maternal infection (all P values > 0.34, Fig. 4a–c) nor offspring sex (all P values > 0.47, data not shown) altered short-term spatial reference memory in probe trial 1. However, regardless of sex, offspring born to infected dams had enhanced long-term spatial reference memory (Fig. 4) as assessed in probe trial 2. Offspring of infected dams had lower *latency* (P = 0.0044; Fig. 4a), *path length* (P = 0.0067; Fig. 4b), and fewer *errors* (P = 0.0031; Fig. 4c) in finding the escape hole than offspring of uninfected dams. Furthermore, when controlling for individual performance in probe trial 1, offspring of infected dams retained their memory over the one-week interval whereas the performance of offspring of uninfected dams declined strongly as shown by the positive *change in latency* (P = 0.0067; Fig. 4d), *path length* (P = 0.015; Fig. 4e), and *errors* (P = 0.01; Fig. 4f). Of note, while findings were not influenced by offspring sex (all P values > 0.1, data not shown), independent of maternal infection, long-term spatial reference memory was positively associated with offspring length (all P values < 0.0015, data not shown). Thus, despite offspring of infected dams being significantly shorter, they outperformed offspring of uninfected dams.

## Discussion

Using a nematode parasite that remains in the maternal intestine, we tested our hypothesis that maternal infection during pregnancy and lactation would positively influence the spatial behavior of pre-weaned and juvenile uninfected male and female offspring. We report for the first time that PD 16 offspring of *H. bakeri* infected dams exhibit less exploratory behaviour compared to pups of uninfected dams, possibly reflecting transient fear of an unfamiliar environment and/or a brief developmental delay. However, in response to maternal infection, PD 17 offspring exhibited better retention of object location memory and at PD 34 they had enhanced long-term spatial reference memory. These novel findings indicate that a maternal GI nematode infection during pregnancy and lactation positively influences the spatial memory of uninfected juvenile mice, despite the negative impact of maternal infection on the linear growth of the pup.

Findings from the OFT indicate that on first introduction to an open arena, offspring of *H. bakeri* infected mothers explored less compared to offspring of uninfected mothers, raising the possibility of a developmental delay and/or heightened fear or anxiety^17,26^. As spontaneous exploration in an open field is first detectable between PD 16–19^17,26^, it is possible that some component of neurodevelopment is delayed at PD 16 in response to maternal infection, which may have negative consequences for the offspring. However, this lower exploration was not observed one day later when these pups were placed in the open field with two novel objects, suggesting that if a developmental delay did occur, it was brief, and may not have had consequences for the growing pup. These findings from the OFT are similar to other maternal infection studies as exposure of pregnant mice to *E. coli*^53^ or influenza virus^25^ resulted in heightened anxiety-like behaviours albeit in adolescent (5 week old)^53^ and adult (9 month old)^25^ offspring, indicated by less exploratory behavior in an OFT. While a heightened fear/anxiety response can be considered a negative attribute, under some circumstances it can be advantageous to the host^3^. Fear and anxiety act as a response to danger or threat^54^, thus when exposed to an unknown environment, mammals typically freeze as it is more difficult for a predator to observe a non-moving animal^26^. Considering that wild rodents are exposed to a number of natural predators, the lower exploration in the open field arena in response to maternal infection may indicate more caution when placed in an unknown and potentially dangerous environment which could actually be beneficial to survival.

The ability to recognize and remember the spatial characteristics of the environment, such as the location of objects, is an important component of spatial cognition^9,19^. This typically begins in 16–17 day old rodents with memory lasting only for a few minutes^18,29^, but for a few hours in 21–24 day old subjects^18,30^. Thus, our observation that PD 17 pups of uninfected mothers were unable to detect object rearrangement after a three hour period was consistent with the literature and suggests a normal immaturity in recalling spatial information at PD 17^55^. However, despite their young age we found that PD 17 pups of *H. bakeri* infected mothers were able to retain object location memories for three hours, as evidenced by a significant increase in investigation of an object after it had been moved. This finding is in contrast with reports that exposure of pregnant rodents to viral mimics had no influence on offspring object location memory, although the studies were done using adult offspring^56,57^. Our findings indicate that the maturational process needed to recall object location memories for three hours occurred earlier as a result of maternal *H. bakeri* infection. This is consistent with recent findings that maternal *H. bakeri* infection up-regulated expression of genes associated with LTP in brains of perinatal uninfected offspring^48^ and thus may promote cognitive development.

The ability to learn the route to an escape location is detectable at PD 21 in rodents^17,19,32,33^, however, long-term reference memories for an escape location in the Morris water maze do not emerge until much later^32,33^. The Morris water maze is similar to the BMT as it assesses spatial learning and reference memory by testing the ability of a subject to locate a hidden underwater platform in order to escape from water in a circular water tank^9^. When PD 20, 34 and 60 subjects were tested in a Morris water maze, all age groups were capable of learning the route to the escape location, and remembering this location for one-day^33^. However, PD 20 and 34 rodents were not yet capable of retaining long-term reference memories for a one-week period whereas PD 60 subjects were^33^. We assessed spatial learning over four days from PD 23–26, followed by short-term reference memory one day later at PD 27 and finally long-term reference memory one-week later at PD 34. Maternal infection had no impact on spatial learning or short-term reference memory, but long-term reference memory was enhanced as a result of this maternal infection. The ability of the juvenile control pups to learn the location of the escape box and recall this location after one day but not one week was consistent with studies using the Morris water maze^32,33^. Unlike control pups, offspring from infected mothers were capable of retaining long-term reference memories for a week as they performed equally well after the one-week delay, compared with the one-day delay. These findings are in the opposite direction to reports from maternal *E. coli* infection models where exposure of pregnant rodents impaired offspring spatial learning and short and long-term reference memory in the Morris water maze^23,24,35^. Overall, our findings reinforce our observation from the OLT that the maturational processes required for the retention of spatial memories occur earlier as a result of this maternal infection and lead us to speculate that maternal *H. bakeri* infection may increase the fitness of the next generation.

Some evidence of sex dependent differences in spatial learning and memory of offspring has been reported in response to prenatal infection mimics whereby molecules of pathogens are injected into the pregnant dam^34–36^. For example, exposure of pregnant rats to *E. coli* lipopolysaccharide (LPS) impaired spatial learning and reference memory in the Morris water maze in 28-day-old male but not female offspring^35^. The underlying mechanisms are unknown, although sex hormones might play a role^35,37,42^. Other studies have shown no impact of offspring sex on spatial behaviour in response to prenatal infection mimics^58,59^. Our results using a direct nematode infection of pregnant mice are consistent with these latter studies in that offspring sex did not affect spatial exploration by offspring in the OFT, their ability to retain object location memories in the OLT, or to learn or remember the escape location in the BMT. Similarly, in the absence of maternal stress, no difference in the spatial behaviour and memory performance was observed between male and female pre-weaned (PD 17–18) CD-1 mice and rats in an OFT and OLT^18,55^, nor between juvenile (PD 22) male and female mice in a Morris water maze test^60^.

The observed impact of maternal infection on spatial learning and memory of their pups could be an indirect consequence of infection-induced nutrient deficiencies but evidence from the literature suggests that this is unlikely. Unlike hookworms that feed on blood and can lead to iron deficiency anemia and protein deficiency when in high numbers^61^, adult *H. bakeri* feed on the epithelial cell layer of the small intestine and are not typically associated with blood loss^62^. Despite lower maternal food intake in response to *H. bakeri* infection during pregnancy^63^, we found no impact of infection on maternal body mass during pregnancy or lactation or on the date of delivery or litter size, all of which would be expected consequences of maternal malnutrition^46,64^. Furthermore, total serum protein concentrations have been shown to be higher in *H. bakeri* infected dams at day 20 of lactation^65^. In the absence of evidence of nutrient deficiencies in pups of *H. bakeri* infected dams and knowing that nutrient deficiencies would be expected to impair not improve spatial memory^66–70^, it is unlikely that nutrient deficiencies account for the improved memory of pups in response to maternal infection.

Maternal *H. bakeri* infection is known to impair fetal^12^ and offspring^46^ linear growth as observed in our study and this impaired growth could have impacted spatial memory. In humans, height, brain size and general cognitive ability are positively correlated^38–41^. Rodent length is correlated with brain size^44^ and brain size has been reported to be a strong indicator of cognitive ability, including the ability to find an escape location in laboratory mice^45^. Our novel finding that mouse length is directly correlated with spatial memory would lead to the expectation that the shorter pups of infected dams would have had impaired spatial memory. However, we found the opposite. Despite their smaller size, it is noteworthy that pups of infected dams were able to recall object locations for 3 h in the OLT and to recall the location of the escape box in the BMT for 1 week, whereas the larger pups of uninfected dams could not.

Formation and retention of spatial memories are controlled in the hippocampus and promoted by LTP and neurogenesis^20,71^. Our observation that offspring of *H. bakeri* infected mothers have enhanced spatial memory is consistent with previous evidence that the brains of PD 7 pups of infected dams have increased expression of LTP genes as well as the ITGA3 gene^48^, which may promote neurogenesis^72^. Further evidence for this hypothesis is found in physical exercise models, where exposure of mice to running enhances hippocampal neurogenesis and LTP which results in enhanced spatial memory performance in the Morris water maze^73,74^. Thus, we speculate that maternal *H. bakeri* infection is capable of enhancing hippocampal LTP and/or neurogenesis in the uninfected pup which promotes the enhanced spatial memory we observed. Further studies would be needed to explore this hypothesis.

The mechanism whereby a nematode living in the lumen of the maternal intestine could influence brain gene expression and alter cognitive processes which promote the spatial memory ability of offspring is unknown. One possibility is that the Th2/Treg immune response in the infected dam^75^ induces a similar systemic response in the uninfected pup that extends to and alters the immune profile in the pup brain. Consistent with this, maternal *H. bakeri* infection up-regulated expression of Th2/Treg pathways and their associated cytokines including interleukin (IL)-4 and transforming growth factor-β (TGF-β) in the PD 7 pup brain, while down-regulating Th1 pathways and the inflammatory cytokine IL-1β^48,76^. Elevated IL-1β has been shown to impair spatial memory^77^, and knock-out studies have highlighted the beneficial and critical importance of IL-4 for the formation and retention of spatial memories^78,79^. Performance of spatial tasks leads to the accumulation of IL-4 producing Th2 cells in the meninges, and deficiency of IL-4 results in severely impaired performance of spatial memory tasks^78^. IL-4 stimulates astrocytes to produce brain-derived neurotrophic factor (BDNF)^78^, a key molecule for regulating cognitive processes, including LTP and neurogenesis^80,81^. Of note, in addition to up-regulating IL-4 expression^48,76^, maternal *H. bakeri* infection also up-regulated BDNF expression in the brains of PD 7 neonates (unpublished data). Therefore we hypothesize that the enhanced spatial memory in the pups of infected dams is associated with a regulatory Th2/Treg neuroimmune environment which promotes LTP and neurogenesis via the production of BDNF by astrocytes. Consistent with our hypothesis that a maternal helminth infection is capable of altering the neuroimmune environment of offspring, Williamson et al.^82^ found that maternal infection with *H. diminuta* blunted the normal increase in hippocampal IL-1β mRNA response to LPS injection in PD 4 offspring. Similar to *H. bakeri, H. diminuta* infects the small intestine and induces a Th2/Treg immune response^83^. Further research is needed to determine whether the Th2/Treg bias is reflected in the neuroimmune environment of the uninfected pup.

We acknowledge four limitations. First, given our hypothesis that spatial memory may emerge earlier due to this maternal infection, we needed to test pre-weaned mice in the OLT, but some of them did not meet our inclusion criterion as they did not explore either object. This was expected as pups would likely have a high level of anxiety and fear due to being separated from their mothers for the test, leading to freezing events and a complete absence of exploration of the arena and objects. Although this lowered our sample size, we had sufficient pups that did explore to be able to detect significant differences between pups of infected and uninfected mothers. Second, we did not determine brain mass of the pups which may have been a more direct covariate for behavioural variables than body length. Third, despite the evidence for improved spatial memory, this maternal GI nematode infection may have negative (or positive) implications on other aspects of brain function and behaviour. Fourth, as our study was focused on the development of spatial cognition in young offspring, our findings cannot be extrapolated to adult mice. Future studies are needed to determine if this maternal GI nematode infection has positive long-term influences on brain development and behavior of the next generation.

To the best of our knowledge, this is the first study to assess the impact of a maternal GI nematode infection on the spatial behaviour of offspring, and to demonstrate enhanced spatial memory in pre-weaned and juvenile uninfected offspring. These findings shed light on a possible unappreciated benefit of maternal GI nematode infection and highlight a possible increase in fitness of the next generation. It would be important to determine if this behavioural impact persists as mice mature and how this maternal infection influences other aspects of offspring behaviour.

## Methodology

### Experimental design

We employed a 2 × 2 factorial design using *H. bakeri* infected versus uninfected dams, and their male versus female offspring.

### Mice and parasites

**Figure 5:**
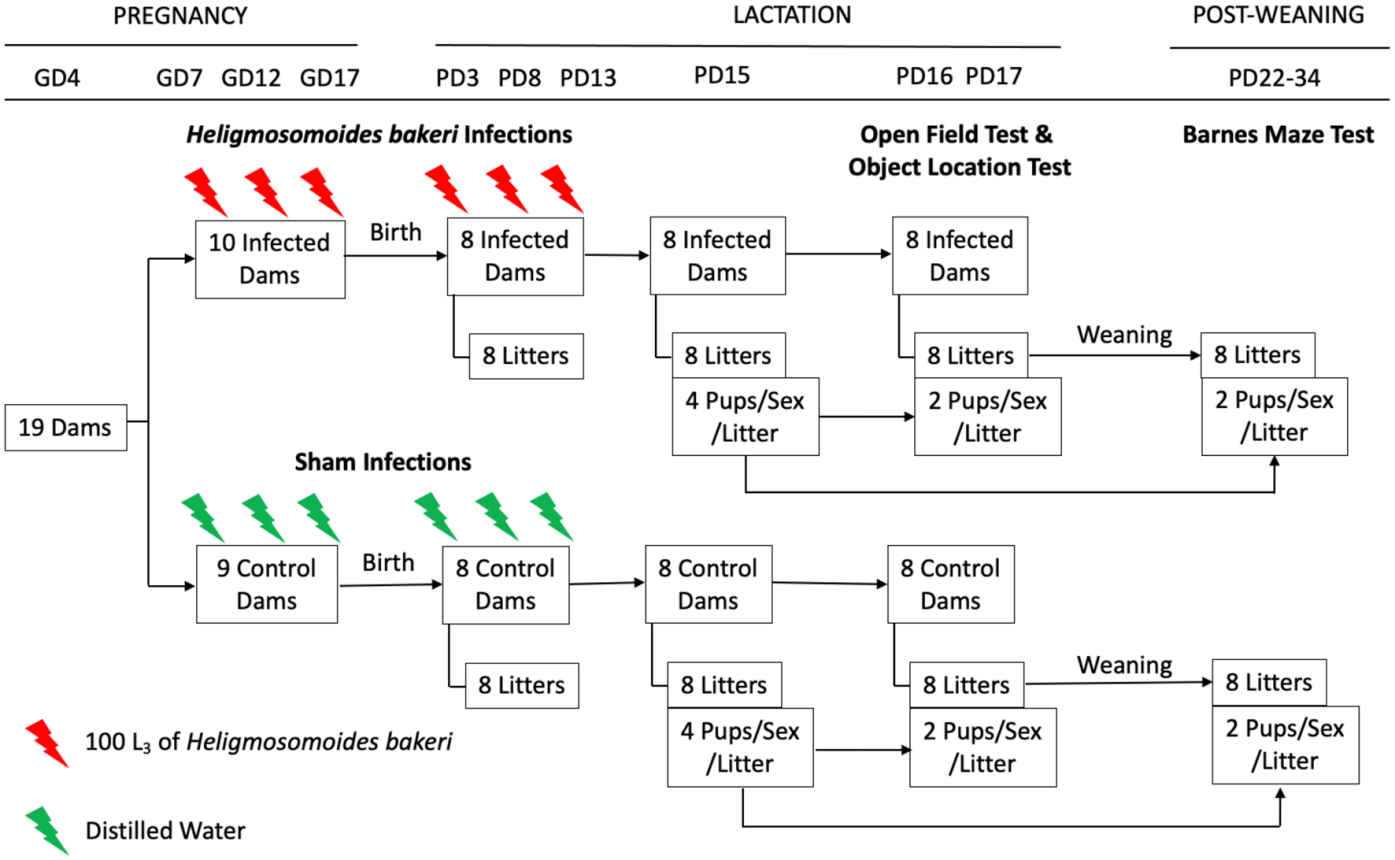
Schematic representing experimental design and protocol. Of the 19 timed-pregnant dams received on gestation day (GD) 4, only 16 delivered litters. On postnatal day (PD) 15, four pups per sex per litter were selected to perform behaviour tests, with half performing the Open Field Test and Object Location Test, and the other half performing the Barnes Maze Test. Of the pups selected for behavioural analysis, their size, specifically crown-rump length and weight, were recorded on PD 15 and 21.

Of the 19 primiparous 8-week-old timed pregnant (gestation day [GD] 4) outbred CD-1 mice (Charles River Laboratories, Quebec, Canada), 16 were pregnant (84% pregnancy rate). Each dam and her litter was housed individually in a Nalgene cage (Fisher Scientific, Canada) at 21–23 °C, 40–60% relative humidity and a 12 h light and dark cycle. Mice had ad libitum access to a 2920X Teklad rodent diet (18% crude protein, 5% crude fat, 5% crude fiber). Within each of the seven staggered groups of dams received over 5 months, dams were randomized into uninfected and infected groups, and a total of eight dams per group were used for this study, providing an acceptable sample size based on a minimum of at least six dams per treatment condition^84^. Using standard *H. bakeri* protocols^85^, infective L_3_ were obtained by fecal culture of stock parasites maintained in outbred CD-1 mice. Dams in the infected group were intubated using an oral gavage needle with 100 ± 3 L_3_ suspended in 0.1 mL distilled water on GD 7, 12, 17, and PD 3, 8 and 13 (Fig. 5). Uninfected dams were intubated at the same frequency with 0.1 mL distilled water. Given that *H. bakeri* eggs released into the environment develop into infective larvae after 7 days, all cages were cleaned every 5 days to ensure offspring could not ingest infective larvae. Successful infection of dams was confirmed through faecal egg counts at weaning (PD 21), and worm counts 13–32 days after weaning (235.4 ± 45.4 worms/dam). Dams were then used for a separate experiment.

Pups were born on GD 19 or 20, litter size was recorded on PD 3, 8, 13, 15 and 21, and body mass and length from the top of the head to the base of the tail were recorded on PD 15 and 21. At PD 15, pups were sexed and given a unique identifier with a permanent marker. Pups were randomly selected to provide two male and two female pups per litter for the OFT/OLT and two male and two female pups per litter for the BMT (Fig. 5). At weaning, pups were separated by sex and 3–4 littermates were housed per cage. After the OFT/OLT or the BMT, experimental pups were necropsied and intestines were examined for larval and adult *H. bakeri*^85,86^. This confirmed that the pups had not been accidentally infected. Pups not used for this study were assigned to a separate experiment.

### Compliance with guidelines for research with experimental animals

This study (protocol #2000–4601) was approved by the McGill University Animal Care Committee according to the guidelines of the Canadian Council on Animal Care. All methods were carried out in accordance with relevant guidelines and regulations, and the study was carried out in compliance with ARRIVE guidelines (https://arriveguidelines.org).

### Experimental room and procedures

All spatial tests were conducted in a quiet room (340 cm × 260 cm) with a floor lamp in each corner that provided dim, even illumination to minimize stress of young pups during the OFT and OLT. During the BMT, a bright over-head light was added to provide a mild negative reinforcement. Trials were recorded using an overhead monochromatic video camera (Basler Ace monochrome) connected to a computer that was located in the back corner of the room behind a curtain. The experimenter remained behind the curtain during all recordings. Data was extracted from the videos using the Ethovision XT software (version 15). All equipment remained in the same location in the room, providing visual spatial cues.

To reduce handling anxiety, each pup in every litter was allowed to explore the palm of the experimenter for two minutes on PD 14 and 15, in their home room. Home cages were moved into the experimental room for 15–20 min acclimation prior to trials and all equipment was cleaned with 70% ethanol between trials.

### Open field test (OFT) and object location test (OLT)

**Figure 6:**
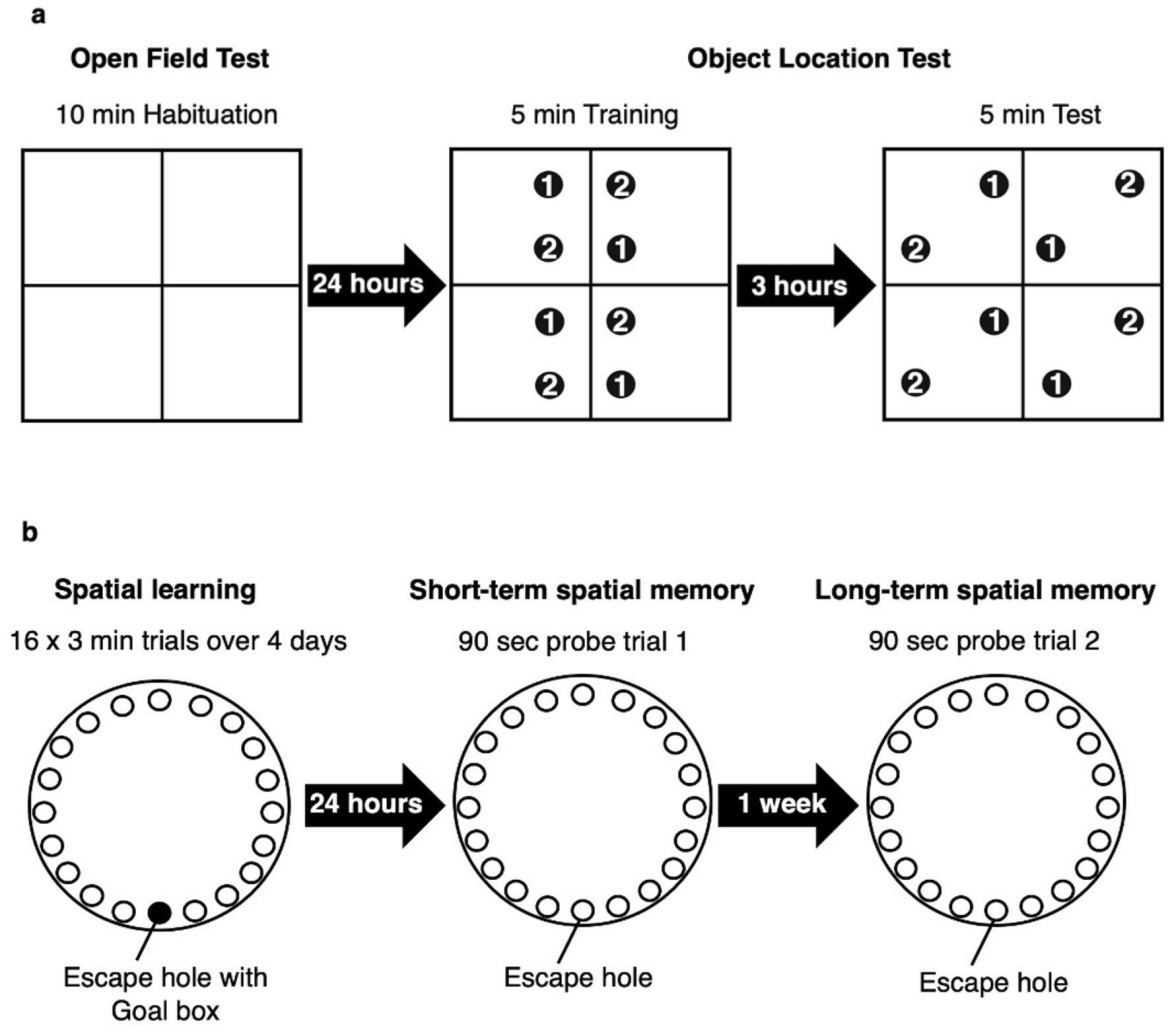
Bird’s eye view of the experimental apparatus and protocols for the Open Field Test and Object Location Test, and the Barnes Maze Test. (**a**) Arena used for both Open Field Test and Object Location Test. Subjects were assigned to one of four identical plexiglass compartments. Two identical objects (Object 1, stationary object and Object 2, mobile object) were added to each compartment after the Open Field Test for the object location training, and the mobile object was moved to a novel location prior to the test trial. Subjects that recognize that Object 2 was in a different location are expected to increase their investigation of this object during the test trial. (**b**) Barnes Maze with 20 equally spaced holes, one of which is the escape hole with a goal box beneath it during the training but not the two probe trials. During training, subjects learn the location of the escape hole relative to spatial cues which surround the maze. Short term spatial reference memory was assessed in probe trial 1, 1 day after training, and long-term spatial reference memory was assessed during probe trial 2, 1 week later.

The OFT/OLT arena (Maze engineers, 412 Wilmette Ave, Glenview, IL 60025, USA) (80 × 80 × 30 cm) had four opaque plexiglass compartments (40 × 40 × 30 cm) that allowed us to test the four pups per litter at the same time (Fig. 6a). An environmental cue (a large cross in colored tape) was placed on an inside wall of each compartment.

The OFT, conducted 24 h prior to the OLT, is an important component of the OLT protocol as it allows the pups to habituate to the novel arena. It also provides information on their exploratory behaviour^28^. On PD 16, pups were introduced to a designated compartment of the arena, and their activity was recorded for 10 min^53^. A preference to stay close to the walls of the field along with freezing behavior (not moving) indicates decreased spatial exploration and increased anxiety-like behavior^26^. For the purpose of data collection, the arena was conceptually partitioned into the peripheral zone (5.86 cm from each wall, totaling 50% of the surface area), and the center zone occupying the remaining area. Four descriptive displacement variables were measured to assess activity: *total path traveled (cm), mean velocity (cm/s), time without movement (%),* and *time in center zone (%)*.

The OLT tested object location memory based on exploration of an object that had been moved to a novel location^27,28,88^ between the 5 min training trial and the 5 min test trial. Pilot testing confirmed that our multi-colored metal cylindrical aerosol cans (diameter: 4 cm, height: 15 cm) were suitable objects for the OLT because young pups did not fear them or climb on, sit on top of, or tip them over. The OLT was conducted on PD 17 using the same mice that had been habituated to the arena during the OFT on PD 16. Prior to the OLT training trial, two identical objects were positioned at designated locations within each compartment (Fig. 6a). Each pup was placed in the compartment as far as possible from both objects to avoid any position bias, and behaviour was video-recorded for 5 min after which pups were returned to their home cage. Prior to the test trial, one of the two objects (Object 2) in each compartment was moved to a novel location (Fig. 6a). A 3 h interval between training and test was selected given that 5 week old CD-1 mice have been shown to retain object location memories after 2 h but not after 4 h^88^, and we had hypothesized that spatial memory would be enhanced in response to maternal infection. Pups were returned to the same compartment for a 5 min test trial. Three descriptive displacement variables were measured during both the training and test trials: the *total path traveled (cm), mean velocity (cm/s),* and *time without movement (%)*. In addition, one exploration variable was recorded for both Object 1 (stationary object) and Object 2 (mobile object): *object investigation time (s)* which measured how long a subject’s nose was within a one-cm radius of the respective Object.

Pups were excluded from analysis of the OLT if they did not explore objects during either the training or test trial (6 males and 2 females from the uninfected group; 6 males and 8 females from the *H. bakeri* group).

### Barnes maze test

The BMT procedure followed a protocol^31^ that successfully tested spatial learning and short and long-term reference memory in CD-1 mice^88^. The Barnes Maze (Maze engineers, 412 Wilmette Ave, Glenview, IL 60025, USA) is an opaque circular platform (diameter: 92 cm, height: 70 cm) with 20 equally spaced holes (diameter: 5 cm) located 2 cm from the edge (Fig. 6b). In a brightly lit environment, mice naturally seek the dark enclosed area provided by the black goal box (20 × 10 × 4 cm) which was located under the same escape hole throughout all trials (Fig. 6b). From the surface of the maze, the escape hole, containing the goal box, looks identical to the other 19 holes. Mice learn the location of the goal box based on spatial cues.

The BMT was conducted on pups that had not been tested in the OFT/OLT. It involved a habitation phase of 5 min on PD 22 (Day 0), a training phase from PD 23–26 (Day 1–4), and probe trials 1 and 2 to test short-term and long-term spatial reference memory on PD 27 (Day 5) and 34 (Day 12) respectively. Training involved four 3 min trials per day for four training days. Each of the 16 training trials began by placing a pup in an opaque starting cylinder (diameter: 10.5 cm, height: 8 cm) at the center of the platform. After 10 s, the cylinder was removed, recording began, and the animal was allowed to freely explore the apparatus for 3 min. Once the animal entered the goal box, it was allowed to remain there for 60 s. Mice that failed to find the goal box within 3 min were gently guided to its location and placed inside. After each of the four 3 min training trials per day, mice were returned to their home cage for 20 min. Prior to probe trials 1 and 2, the goal box was removed from the escape hole and mice explored the maze for 90 s. No training occurred between the two probe trials.

Variables assessed during all trials were: (1) *latency (s)*, defined as time taken to the first visit (nose poke) to the escape hole; (2) *path length (cm)*, defined as distance travelled to the first visit to the escape hole; and (3) *errors*, defined as number of times a subject visited non-escape holes, before their first visit to the escape hole. *Mean velocity (cm/s)* during the training trials was used to determine if performance differences reflected motor ability that may have been influenced by pup length.

### Statistical analyses

Statistical analyses were performed in R statistical software 4.0.2^89^, and figures were produced using the package ggplot2^90^. Maternal treatment condition (*H. bakeri* infected versus uninfected) and offspring sex (male versus female) were always included as fixed factors. For comparisons over time, trial was included as a fixed factor. To account for pseudoreplication, dam was a random factor in all models, and the identity of the pup was also included as a random factor for comparisons over time where we had repeated measures on pups^91^. Non-significant interactions between fixed effects were excluded from models^92^. Pup length was included as a covariate in all models of behaviour data.

Linear mixed models (LMMs) or Generalized linear mixed models (GLMMs) were built using the lmer or glmer function, respectively (lme4 package^93^), with significance assessed using the Anova function (car package^94^). Where necessary, post hoc pairwise comparisons were performed using the emmeans function (emmeans package^95^) with a Tukey correction. Normality, independence and homogeneity of variances of mixed models were assessed using fitted residuals from the plotresid function (RVAideMemoire package^96^), and in the case of GLMMs, also using the DHARMa package^97^. Unless otherwise stated, values are presented as LSmeans ± SEM from the emmeans function. The significance level was set at 0.05.

As no pup mortality occurred, the influence of the maternal infection status on litter size was analyzed on PD 21 using a linear model (lm function^89^). LMMs were used to compare pup length and mass at PD 15 and PD 21 between experimental groups, with litter size as a covariate.

OFT/OLT: LMMs were used to assess object bias, displacement and exploration variables in the OFT and OLT. Two additional derivative variables were calculated from the OLT data to assess object location memory and both were analysed by LMM. The *% investigation time of mobile object* was used to compare the investigation of the mobile object relative to the total time spent investigating both objects in both the training and test trial, and calculated as: [Object 2 (mobile) investigation time (s) / [Object 1 (stationary) investigation time (s) + Object 2 (mobile) investigation time (s)]] *100. This variable ranged from 0% (only investigated Object 1) to 100% (only investigated Object 2). The *change in % investigation time of mobile object* allowed us to control results from the test trial with individual performance during the training trial, and was calculated as: % investigation time of mobile object during the test trial—% investigation time of mobile object during the training trial. A positive value indicated that the subject explored Object 2 more during the test trial compared to the training trial, indicating an increased investigation in the object after it had been moved.

BMT: Data in the BMT were positively skewed, and in some instances, heteroscedastic. The best distribution was assessed using the functions descdist and fitdist (package fitdistrplus^98^) and comparing model residuals for best fit. In the training phase, we used LMM with log transformations for *latency, path length* and *mean velocity*. In the probe trials, we used Gamma GLMM, with log link function, for *latency* and *path length*. The number of *errors* was a discrete and overdispersed variable, and a negative binomial GLMM, with log link function, was used for both the training and probe trials.

In addition, a set of derivative variables reflecting *change in latency, path length* and *errors* between the two probe trials was calculated by subtracting probe trial 1 values from probe trial 2 values, allowing us to control for individual performance during probe trial 1. These derivative variables were normally distributed and homoscedastic, and LMMs were used without transformation.

## Supplementary Information

Supplementary Figure 1.

Supplementary Information 2.

## Data Availability

The authors confirm that the data supporting the findings of this study are available as supplementary material S1.

## References

[1] Zaiss MM, Harris NL (2016). Interactions between the intestinal microbiome and helminth parasites. Parasite Immunol..

[2] Jhan KY (2020). *Angiostrongylus cantonensis* causes cognitive impairments in heavily infected BALB/c and C57BL/6 mice. Parasites Vectors..

[3] Boillat M (2020). Neuroinflammation-associated aspecific manipulation of mouse predator fear by *Toxoplasma gondii*. Cell Rep..

[4] Brombacher TM (2018). *Nippostrongylus brasiliensis* infection leads to impaired reference memory and myeloid cell interference. Sci. Rep..

[5] Kavaliers M, Colwell DD (1995). Reduced spatial learning in mice infected with the nematode Heligmosomoides polygyrus. Parasitology.

[6] Pan SC (2019). Cognitive and microbiome impacts of experimental *Ancylostoma ceylanicum* hookworm infections in hamsters. Sci. Rep..

[7] Blecharz-Klin K (2022). Infection with intestinal helminth (*Hymenolepis diminuta*) impacts exploratory behavior and cognitive processes in rats by changing the central level of neurotransmitters. PLoS Pathog..

[8] Braithwaite V (1998). Spatial and discrimination learning in rodents infected with the nematode *Strongyloides ratti*. Parasitology.

[9] Sharma S, Rakoczy S, Brown-Borg H (2010). Assessment of spatial memory in mice. Life Sci..

[10] Vorhees CV, Williams MT (2014). Assessing spatial learning and memory in rodents. ILAR J..

[11] Pelletier F, Page KA, Ostiguy T, Festa-Bianchet M (2005). Fecal counts of lungworm larvae and reproductive effort in bighorn sheep. Ovis canadensis. Oikos..

[12] Odiere MR, Koski KG, Weiler HA, Scott ME (2010). Concurrent nematode infection and pregnancy induce physiological responses that impair linear growth in the murine foetus. Parasitology.

[13] Fitzgerald E, Hor K, Drake AJ (2020). Maternal influences on fetal brain development: The role of nutrition, infection and stress, and the potential for intergenerational consequences. Early Hum. Dev..

[14] Boksa P (2010). Effects of prenatal infection on brain development and behavior: a review of findings from animal models. Brain Behav. Immun..

[15] Akitake Y (2015). Moderate maternal food restriction in mice impairs physical growth, behavior, and neurodevelopment of offspring. Nutr. Res..

[16] Hammelrath L (2016). Morphological maturation of the mouse brain: An *in vivo* MRI and histology investigation. Neuroimage.

[17] Wills T, Muessig L, Cacucci F (2014). The development of spatial behaviour and the hippocampal neural representation of space. Philos. Trans. R. Soc. B: Biol. Sci..

[18] Travaglia A, Steinmetz AB, Miranda JM, Alberini CM (2018). Mechanisms of critical period in the hippocampus underlie object location learning and memory in infant rats. Learn Mem..

[19] McHail DG, Valibeigi N, Dumas TC (2018). A Barnes maze for juvenile rats delineates the emergence of spatial navigation ability. Learn Mem..

[20] Bliss TVP, Collingridge GL, Morris RGM, Reymann KG (2018). Long-term potentiation in the hippocampus: Discovery, mechanisms and function. Neuroforum.

[21] Schiller D (2015). Memory and space: Towards an understanding of the cognitive map. J. Neurosci..

[22] Kim JJ, Diamond DM (2002). The stressed hippocampus, synaptic plasticity and lost memories. Nat. Rev. Neurosci..

[23] Jiang P (2013). The persistent effects of maternal infection on the offspring's cognitive performance and rates of hippocampal neurogenesis. Prog. Neuropsychopharmacol. Biol. Psychiatry..

[24] Wallace, K. L. *et al.* Interleukin-10/Ceftriaxone prevents *E. coli*-induced delays in sensorimotor task learning and spatial memory in neonatal and adult Sprague-Dawley rats. *Brain. Res. Bull.* **81**, 141–148 (2010). 10.1016/j.brainresbull.2009.10.016 PMC2908377 19883741

[25] Shi L, Fatemi SH, Sidwell RW, Patterson PH (2003). Maternal influenza infection causes marked behavioral and pharmacological changes in the offspring. J. Neurosci..

[26] Denenberg VH (1969). Open-field behavior in the rat: what does it mean?. Ann. N. Y. Acad. Sci..

[27] Vogel-Ciernia, A. & Wood, M. A. Examining object location and object recognition memory in mice. *Curr. Protoc. Neurosci.* **69**, 8.31.1–17 (2014). 10.1002/0471142301.ns0831s69 PMC4219523 25297693

[28] Denninger JK, Smith BM, Kirby ED (2018). Novel object recognition and object location behavioral testing in mice on a budget. J. Vis. Exp..

[29] Krüger H-S, Brockmann MD, Salamon J, Ittrich H, Hanganu-Opatz IL (2012). Neonatal hippocampal lesion alters the functional maturation of the prefrontal cortex and the early cognitive development in pre-juvenile rats. Neurobiol. Learn. Mem..

[30] Cruz-Sanchez A (2020). Developmental onset distinguishes three types of spontaneous recognition memory in mice. Sci. Rep..

[31] Sunyer B, Patil S, Hoger H, Lubec G (2007). Barnes maze, a useful task to assess spatial reference memory in the mice. Nat. Protoc..

[32] Schenk F (1985). Development of place navigation in rats from weaning to puberty. Behav. Neural Biol..

[33] Brown RW, Kraemer PJ (1997). Ontogenetic differences in retention of spatial learning tested with the Morris water maze. Dev. Psychobiol..

[34] Batinić B (2016). Lipopolysaccharide exposure during late embryogenesis results in diminished locomotor activity and amphetamine response in females and spatial cognition impairment in males in adult, but not adolescent rat offspring. Behav. Brain Res..

[35] Lante F (2007). Neurodevelopmental damage after prenatal infection: role of oxidative stress in the fetal brain. Free Radic. Biol. Med..

[36] Wang H (2010). Age- and gender-dependent impairments of neurobehaviors in mice whose mothers were exposed to lipopolysaccharide during pregnancy. Toxicol. Lett..

[37] Yagi S, Galea LAM (2019). Sex differences in hippocampal cognition and neurogenesis. Neuropsychopharmacology.

[38] Vuoksimaa E (2018). Brain structure mediates the association between height and cognitive ability. Brain Struct. Func..

[39] Harris MA, Brett CE, Deary IJ, Starr JM (2016). Associations among height, body mass index and intelligence from age 11 to age 78 years. BMC Geriatr..

[40] Pereira, V. H. *et al.* Adult body height is a good predictor of different dimensions of cognitive function in aged individuals: A cross-sectional study. *Front. Aging Neurosci.* **8**, 1. 10.3389/fnagi.2016.00217 (2016). 10.3389/fnagi.2016.00217 PMC5025434 27695413

[41] Case A, Paxson C (2008). Stature and status: Height, ability, and labor market outcomes. J. Polit. Econ..

[42] Frick KM, Kim J, Tuscher JJ, Fortress AM (2015). Sex steroid hormones matter for learning and memory: estrogenic regulation of hippocampal function in male and female rodents. Learn Mem..

[43] Qiu LR (2018). Mouse MRI shows brain areas relatively larger in males emerge before those larger in females. Nat. Commun..

[44] Towe AL, Mann MD (1992). Brain size/body length relations among myomorph rodents. Brain Behav. Evol..

[45] Perepelkina, O. V., Tarasova, A. Y., Ogienko, N. A., Lil’p, I. G. & Poletaeva, I. I. Brain weight and cognitive abilities of laboratory mice. *Biol. Bull. Rev.* **10**, 91–101 (2020).

[46] Odiere MR, Scott ME, Weiler HA, Koski KG (2010). Protein deficiency and nematode infection during pregnancy and lactation reduce maternal bone mineralization and neonatal linear growth in mice. J. Nutr..

[47] Sánchez MB (2021). *Leishmania amazonensis* infection impairs reproductive and fetal parameters in female mice. Rev. Argent. Microbiol..

[48] Haque M, Koski KG, Scott ME (2019). Maternal gastrointestinal nematode infection up-regulates expression of genes associated with long-term potentiation in perinatal brains of uninfected developing pups. Sci. Rep..

[49] Gregory RD, Montgomery SSJ, Montgomery WI (1992). Population biology of *Heligmosomoides polygyrus* (Nematoda) in the wood mouse. J. Anim. Ecol..

[50] Reynolds LA, Filbey KJ, Maizels RM (2012). Immunity to the model intestinal helminth parasite *Heligmosomoides polygyrus*. Semin. Immunopathol..

[51] Maizels RM (2012). Immune modulation and modulators in *Heligmosomoides polygyrus* infection. Exp. Parasitol..

[52] Haque M, Starr LM, Koski KG, Scott ME (2018). Differential expression of genes in fetal brain as a consequence of maternal protein deficiency and nematode infection. Int. J. Parasitol..

[53] Hsueh, P.-T. *et al.* Immune imbalance of global gene expression, and cytokine, chemokine and selectin levels in the brains of offspring with social deficits via maternal immune activation. *Genes Brain Behav.* **17**, e12479. 10.1111/gbb.12479 (2018). 10.1111/gbb.12479 29656594

[54] Steimer T (2002). The biology of fear- and anxiety-related behaviors. Dialogues Clin. Neurosci..

[55] Ricceri L, Colozza C, Calamandrei G (2000). Ontogeny of spatial discrimination in mice: A longitudinal analysis in the modified open-field with objects. Dev. Psychobiol..

[56] Howland JG, Cazakoff BN, Zhang Y (2012). Altered object-in-place recognition memory, prepulse inhibition, and locomotor activity in the offspring of rats exposed to a viral mimetic during pregnancy. Neuroscience.

[57] Ito HT, Smith SEP, Hsiao E, Patterson PH (2010). Maternal immune activation alters nonspatial information processing in the hippocampus of the adult offspring. Brain Behav. Immun..

[58] Meyer U (2006). The time of prenatal immune challenge determines the specificity of inflammation-mediated brain and behavioral pathology. J. Neurosci..

[59] Meyer U (2008). Adult behavioral and pharmacological dysfunctions following disruption of the fetal brain balance between pro-inflammatory and IL-10-mediated anti-inflammatory signaling. Mol. Psychiatry..

[60] Chapillon P, Roullet P (1996). Use of proximal and distal cues in place navigation by mice changes during ontogeny. Dev. Psychobiol..

[61] Variyam EP, Banwell JG (1982). Hookworm disease: Nutritional implications. Rev. Infect. Dis..

[62] Bansemir AD, Sukhdeo MV (1994). The food resource of adult *Heligmosomoides polygyrus* in the small intestine. J. Parasitol..

[63] Starr LM, Scott ME, Koski KG (2015). Protein deficiency and intestinal nematode infection in pregnant mice differentially impact fetal growth through specific stress hormones, growth factors, and cytokines. J. Nutr..

[64] Herring CM, Bazer FW, Johnson GA, Wu G (2018). Impacts of maternal dietary protein intake on fetal survival, growth, and development. Exp. Biol. Med. (Maywood)..

[65] Starr LM, Odiere MR, Koski KG, Scott ME (2014). Protein deficiency alters impact of intestinal nematode infection on intestinal, visceral and lymphoid organ histopathology in lactating mice. Parasitology.

[66] Bastian TW, von Hohenberg WC, Mickelson DJ, Lanier LM, Georgieff MK (2016). Iron deficiency impairs developing hippocampal neuron gene expression, energy metabolism, and dendrite complexity. Dev. Neurosci..

[67] Bastian TW, Rao R, Tran PV, Georgieff MK (2020). The effects of early-life iron deficiency on brain energy metabolism. Neurosci. Insights..

[68] Gould JM (2018). Mouse maternal protein restriction during preimplantation alone permanently alters brain neuron proportion and adult short-term memory. Proc. Natl. Acad. Sci..

[69] Radlowski E, Johnson R (2013). Perinatal iron deficiency and neurocognitive development. Front. Hum. Neurosci..

[70] Rytych JL (2012). Early life iron deficiency impairs spatial cognition in neonatal piglets. J. Nutr..

[71] Snyder JS, Hong NS, McDonald RJ, Wojtowicz JM (2005). A role for adult neurogenesis in spatial long-term memory. Neuroscience.

[72] Brązert M (2020). Expression of genes involved in neurogenesis, and neuronal precursor cell proliferation and development: Novel pathways of human ovarian granulosa cell differentiation and transdifferentiation capability *in vitro*. Mol. Med. Rep..

[73] van Praag H, Christie BR, Sejnowski TJ, Gage FH (1999). Running enhances neurogenesis, learning, and long-term potentiation in mice. Proc. Natl. Acad. Sci. U.S.A..

[74] Li H (2013). Regular treadmill running improves spatial learning and memory performance in young mice through increased hippocampal neurogenesis and decreased stress. Brain. Res..

[75] Odiere MR, Scott ME, Leroux LP, Dzierszinski FS, Koski KG (2013). Maternal protein deficiency during a gastrointestinal nematode infection alters developmental profile of lymphocyte populations and selected cytokines in neonatal mice. J. Nutr..

[76] El Ahdab N, Haque M, Madogwe E, Koski KG, Scott ME (2021). Maternal nematode infection upregulates expression of Th2/Treg and diapedesis related genes in the neonatal brain. Sci. Rep..

[77] Hein AM (2010). Sustained hippocampal IL-1β overexpression impairs contextual and spatial memory in transgenic mice. Brain Behav. Immun..

[78] Derecki NC (2010). Regulation of learning and memory by meningeal immunity: A key role for IL-4. Exp. Med..

[79] Brombacher TM (2020). IL-4R alpha deficiency influences hippocampal-BDNF signaling pathway to impair reference memory. Sci. Rep..

[80] Mizuno M, Yamada K, Olariu A, Nawa H, Nabeshima T (2000). Involvement of brain-derived neurotrophic factor in spatial memory formation and maintenance in a radial arm maze test in rats. J. Neurosci..

[81] Miranda, M., Morici, J. F., Zanoni, M. B. & Bekinschtein, P. Brain-derived neurotrophic factor: A key molecule for memory in the healthy and the pathological brain. *Front. Cell. Neurosci.* **13**. 10.3389/fncel.2019.00363 (2019). 10.3389/fncel.2019.00363 PMC6692714 31440144

[82] Williamson LL (2016). Got worms? Perinatal exposure to helminths prevents persistent immune sensitization and cognitive dysfunction induced by early-life infection. Brain Behav. Immun..

[83] McKay DM (2010). The immune response to and immunomodulation by *Hymenolepis diminuta*. Parasitology.

[84] Meyer U, Feldon J, Fatemi SH (2009). *In-vivo* rodent models for the experimental investigation of prenatal immune activation effects in neurodevelopmental brain disorders. Neurosci. Biobehav. Rev..

[85] Johnston, C. J. C. *et al.* Cultivation of *Heligmosomoides polygyrus*: an immunomodulatory nematode parasite and its secreted products. *J. Vis. Exp.* e52412–e52412. 10.3791/52412 (2015). 10.3791/52412 PMC4401400 25867600

[86] Valanparambil RM (2014). Production and analysis of immunomodulatory excretory-secretory products from the mouse gastrointestinal nematode *Heligmosomoides polygyrus bakeri*. Nat. Protoc..

[87] Murai T, Okuda S, Tanaka T, Ohta H (2007). Characteristics of object location memory in mice: Behavioral and pharmacological studies. Physiol. Behav..

[88] Patil SS, Sunyer B, Hoger H, Lubec G (2009). Evaluation of spatial memory of C57BL/6J and CD1 mice in the Barnes maze, the Multiple T-maze and in the Morris water maze. Behav. Brain. Res..

[89] R: A Language and Environment for Statistical Computing (R Foundation for Statistical Computing, Vienna, Austria, 2020).

[90] Wickham H (2016). ggplot2: Elegant graphics for data analysis.

[91] Lazic SE (2010). The problem of pseudoreplication in neuroscientific studies: Is it affecting your analysis?. BMC Neurosci..

[92] Zuur, A., Ieno, E. N., Walker, N., Saveliev, A. & Smith, G. M. *Mixed effects models and extensions in ecology with R*. Vol. 1–574 (2009).

[93] Bates D, Maechler M, Bolker B, Steve W (2015). Fitting linear mixed-effects models using lme4. J. Stat. Softw..

[94] Fox, J. & Sanford, W. *An R Companion to Applied Regression*. 3 edn, (Sage, 2019).

[95] emmeans: Estimated marginal means, aka least-squares means v. 1.4.8 (R package, 2020).

[96] RVAideMemoire: Testing and plotting procedures for biostatistics. v. 0.9-78 (R package, 2020).

[97] DHARMa: Residual diagnostics for hierarchical (multi-level/mixed) regression models v. 0.3.3.0 (R package, 2020).

[98] Delignette-Muller ML, Dutang C (2015). fitdistrplus: An R package for fitting distributions. J. Stat. Softw..

