# Supplementary material for: Maternal gastrointestinal nematode infection enhances spatial memory of uninfected juvenile mouse pups
Source: Sci Rep. 2022 Jun 13;12:9796. doi: 10.1038/s41598-022-13971-y (PMC9192650; doi:10.1038/s41598-022-13971-y)

Supplementary Figure

Supplementary Figure 1. Effect of maternal *H. bakeri* infection and offspring sex on offspring size at postnatal day (PD) 15 and 21. Pups were nested within dam, and litter size was included as a covariate. Values are LSmeans±SEM, n = 32 male and 32 female pups from 8 uninfected dams and n = 31 male and 33 female pups from 8 infected dams (**$P < 0.01$, ***$P < 0.001$). a) pup crown-rump length and b) pup body mass.

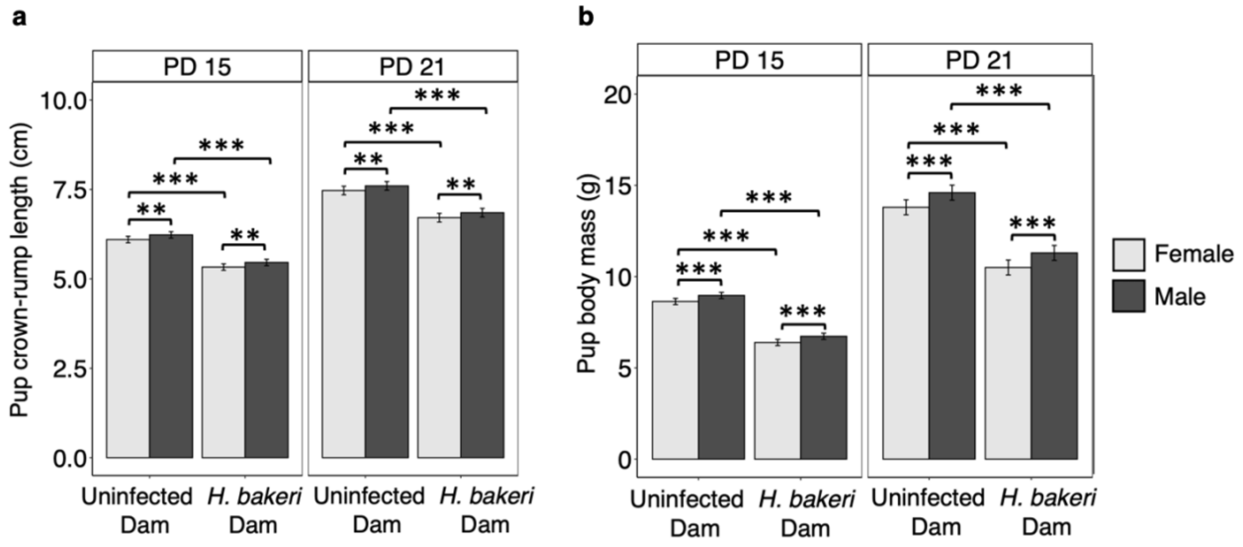

Supplement: Supplementary file 1 — Supplementary Figure 1. [file 41598_2022_13971_MOESM1_ESM.pdf]
